# The dynamic 3D horse: analyzing the relationship between whole body pathomechanics and joint degeneration in the fetlocks

**DOI:** 10.3389/fvets.2026.1773617

**Published:** 2026-03-11

**Authors:** Gwyneth P. Miller, Jean Luc Cornille, Ronda Hanning, Alexander K. K. Lee, Elizabeth W. Uhl, Michelle L. Osborn

**Affiliations:** 1 Department of Comparative Biomedical Sciences, Louisiana State University School of Veterinary Medicine, Baton Rouge, LA, United States; 2 Science of Motion®, Eatonton, GA, United States; 3 Field of Dreams Equestrian Center, LLC, Fort Wayne, IN, United States; 4 Department of Pathology, University of Georgia College of Veterinary Medicine, Athens, GA, United States

**Keywords:** 3D dynamic model, CT data, equine, movement, pathomechanics, posture, rehabilitation, whole-body analysis

## Abstract

Lameness is often associated with degenerative joint disease (DJD). Current therapies focus on minimizing pain or treating specific lesions but generally do not address the pathomechanical forces that are the root cause of degeneration. Treatments based on specific, whole-body therapies are becoming common in humans with DJD, but are still not routinely applied in equine cases. Designing targeted therapies for horses requires recognizing habitual postures and movements that are pathological. An important but often missing component for understanding movement postures is accurate and manipulatable anatomical models. In this case study, a three-dimensional model of a horse based on CT data is manipulated using advanced imaging and animation software Autodesk® Maya® to demonstrate the habitual working posture of a horse with DJD of the fetlock joints before, during, and after the application of a whole-body exercise regime focused on rebalancing the forces negatively impacting the hindlimbs. The horse’s movement postures throughout the targeted therapy are compared by assessing qualitative and quantitative changes in spinal curvature and fetlock joint angles. This case study serves as proof of concept for the feasibility of modeling working postures before and after rehabilitative therapy for the purpose of demonstrating the effects of physical therapy or training programs. It also provides an example of how data obtained from advanced imaging techniques like computed tomography can be used for veterinary medical breakthroughs that are based on rethinking: (1) the relationship between equine posture/movement and pathological conditions of the musculoskeletal system and (2) related training and rehabilitative strategies.

## 1 Introduction

Lameness is the most common diagnosis in equine veterinary medicine, and its presence is often associated with degenerative tissue changes (1–3). In humans, degenerative joint disease (DJD) is an extremely common health problem, but unfortunately, as is the case with horses, there are few effective therapies (4). The lack of effective interventions stems from the fact that most current therapies (e.g., pharmacotherapy and direct chondral repair) focus on minimizing pain or on treating specific lesions (2, 5–7) rather than addressing the pathomechanical forces that are the root cause of the degeneration. The importance of this paradigm shift has been emphasized by the finding that the pathomechanical forces directly impacting both bone and cartilage cells cause degeneration and the release of inflammatory mediators (4). These observations indicate that trying to repair degenerative lesions without correcting the causative mechanical forces is unlikely to be truly effective. As a result, the therapeutic focus in humans is shifting to methods that ‘unload’ the affected joints (8–10). In humans with DJD, unloading joints, especially through targeted exercise, has been so effective in reducing symptoms that many believe reestablishing a favorable mechanical environment will often render pharmacological therapies and invasive joint procedures unnecessary (11).

Comprehensive biomechanical analysis is commonly used to design training programs that facilitate injury recovery in humans (12–14) but is rarely done for horses [see (15)]. Designing such therapies for horses requires the ability to recognize potentially damaging habitual body postures and the understanding that, given its primary role in limb kinematics, the root cause of the lameness likely originates in aberrant movement of the spine rather than the affected limbs (16, 17). Even though there is little scientific evidence connecting conformation to lameness (18), conformation is routinely considered clinically (19, 20). Working body posture, however, is often not considered even though it is even more important in assessing individual pathomechanics (21). While training programs and physical therapies have been designed to address specific issues in horses, documenting their effectiveness is difficult.

This case study serves as proof-of-concept for using a CT data-based, dynamic graphic 3D equine model (22) to investigate the relationship between the whole-body working postures of a competitive Grand Prix dressage horse with degenerative tissue changes of the hindlimb fetlock (metatarsophalangeal) joints. Common whole-body working postures were identified through videos and evaluated using photographs of the horse being worked before, during, and after the implementation of a training approach developed by Science of Motion® (SOM). These postures were modeled, compared, and analyzed using a dynamic 3D equine model. This case study demonstrates that the model is a tool that can be used to analyze posture and movement, and objectively document the effectiveness of training programs. It can also be used to develop re- and pre-habilitative therapies and to assist riders, trainers, and horse enthusiasts in better understanding how the horse moves.

## 2 Materials and methods

### 2.1 Case study details

*History*: 14-year-old Oldenburg Gelding with a history of competing in Grand Prix dressage.

*Diagnostic findings*: In a pre-purchase exam, a veterinarian noted damage in all the fetlock joints, the hind being the most affected, that included decreased range of motion, tendon sheath and joint effusion, thickened and/or sensitive suspensory branches and resistance to flexion.

*Treatment*: Rehabilitation using SOM techniques began at purchase and focused on correcting postural and muscular imbalances throughout the body with the goal being to restore soundness and establish healthy locomotion. The rehabilitation was directed by the founder of SOM (JLC) who has extensive experience in correcting habitual working postures and problematic movement patterns in horses. His assessment of the horse and working hypothesis was that the forward transmission of forces generated by the thrust of the hind legs was being hampered by a rigidness of the thoracic spine.

The fetlock joint allows flexion, extension, and hyperextension, in which the palmar or plantar surface of the fetlock joint gets extremely close to touching the ground (this movement is sometimes referred to as dorsiflexion or dropped fetlock). While poor conformation may pre-dispose the fetlock joint to be routinely in a hyperextended position (i.e., conformational or anatomic hyperextension), the way a horse is exercised may produce the same hyperextended position (i.e., functional hyperextension). In this case, functional hyperextension of the fetlocks was noted in most gaits, but was most extreme when the horse was in Piaffe, a “highly collected, cadenced, elevated” diagonally coordinated gait that gives the impression of the horse prancing in place (23). Because of the routine, extreme hyperextension in this gait, Piaffe was selected for analysis and before and after comparisons. A specific exercise regime was designed that focused on correct and consistent movement at the collected trot. The collected trot emphasizes self-carriage and shorter steps as compared to the “free, active, and regular steps” of the trot (23). It was selected as a focus of therapy because it is less collected and demanding than piaffe and is often used to prepare the horse for proper functioning in the piaffe.

### 2.2 Analysis

**Figure 1:**
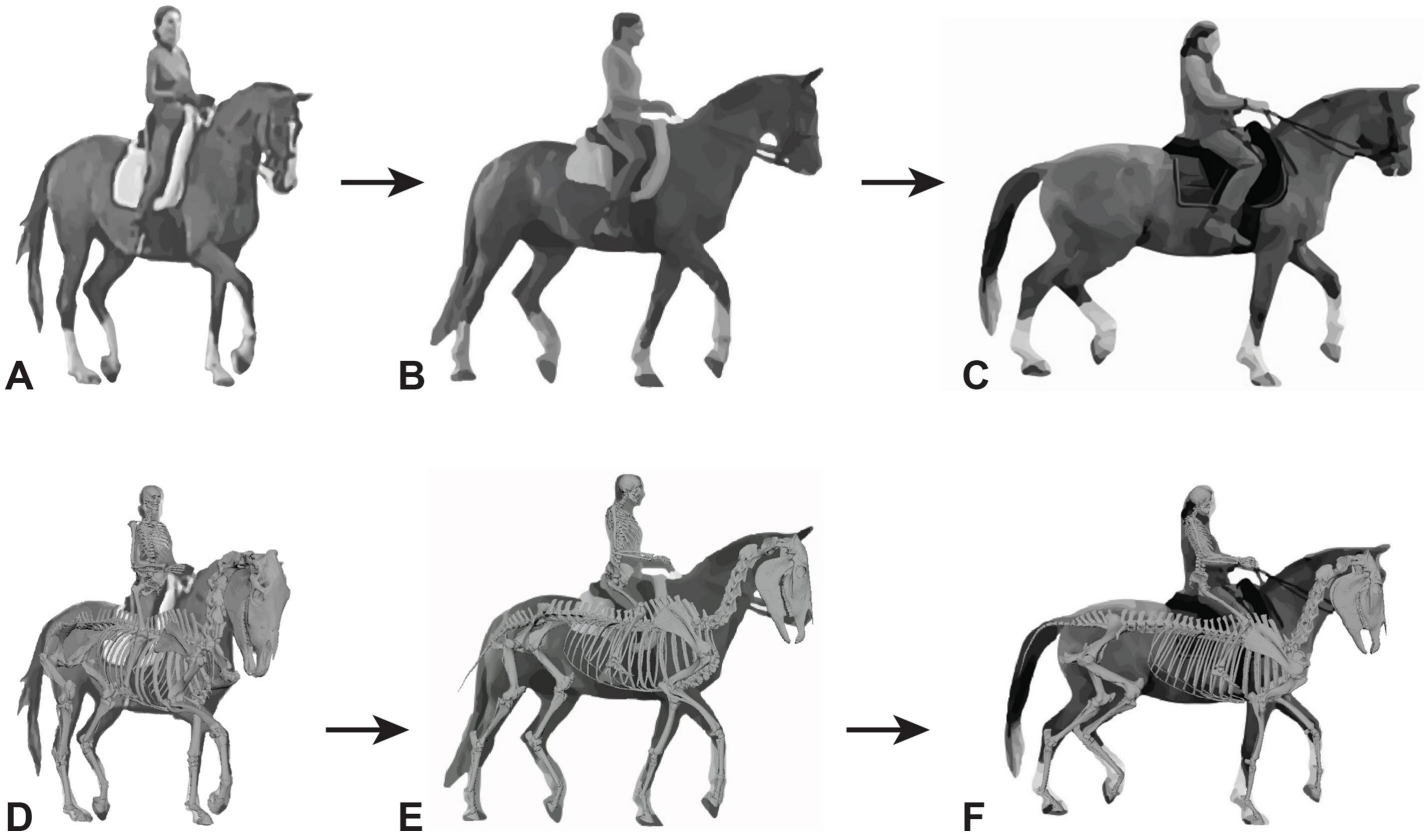
Representative images of the gelding depicting working posture in Piaffe before **(A)** and after **(C)** the implementation of a targeted exercise regime **(B)**. The skeletal model was positioned and superimposed on images of the gelding depicting working posture in Piaffe before **(D)** and after **(F)** the implementation of a targeted exercise regime in the collected trot **(E)**. The actual photographs have been anonymized to protect the identities of the participants. The freeze frame images were selected based on matching the positions of the four limbs and images came from differing perspectives based on camera and horse position.

**Figure 2:**
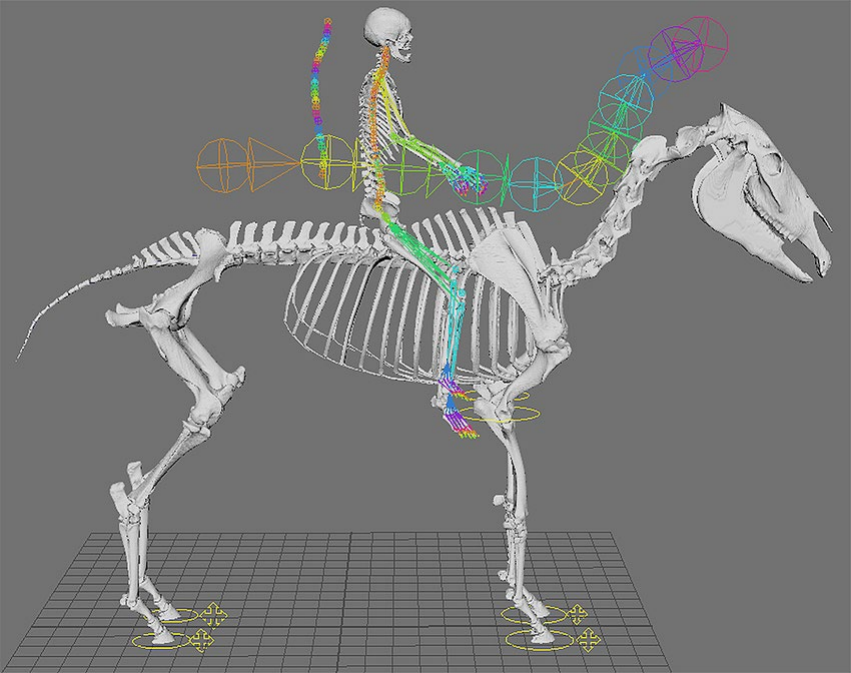
The joints of the 3D equine skeletal model were aligned to each of the postures depicted in the representative images (Figure 1) by rotating and translating skeletal elements using special controls, indicated with vibrant colors in the figure.

Representative images of the gelding depicting its common working posture in Piaffe before, during, and after the implementation of a targeted exercise regime in the collected trot were selected by the trainer from observations of hours of video recordings (Figure 1). The freeze frame images were selected based on matching the positions of the fetlock joints of the four limbs. Although the image was not always in the same perspective (e.g., lateral) because the horse was moving freely while the camera was static on a tripod, the 3D model enables accurate matching of limb positions (Figure 1) and then can be rotated into comparable perspectives (Table 1). The actual photographs were anonymized with Adobe Illustrator to protect the identities of the participants. The animation software Autodesk Maya® was used to position a dynamic 3D model horse skeleton based on computed tomography data (22) to match the horse’s position in each of the representative images (Figure 1). The model was holistically scaled to more closely match the size of the horse in the images. The joints of the 3D equine skeletal model were adjusted using Autodesk Maya® by rotating the spinal controls and rotating and translating the limb controls (Figure 2) to align with the postures depicted in the representative images of the subject horse (Figure 1). The exact placement of the skeleton was based on the easily visible fetlock joints of each limb and, to a lesser extent, the hocks (i.e., tarsi) and knees (i.e., carpi); the other elements were estimated as no implanted or surface markers were used on the horse being exercised. Autodesk Maya® was used to mark specific joint landmarks and to manipulate joint chains to find respective joint angles in the neck and limbs (Supplementary Figures 1–3). The distance measure tool was used to find the distance between several cervical and thoracic vertebrae (Supplementary Figure 3). To measure joint angles in Autodesk Maya®, the joint was first defined with three landmarks. As the limbs were moved, the landmarks moved with them to accurately depict a joint angle in different postures. Autodesk Maya® indicated the degree of change from a straight line (180°), which was then subtracted from 180, thus defining the joint angle. A protractor was used to confirm the angle measurements of the joints as calculated using the method described.

**Table 1:** Joint angles values and intervertebral distances before, during, and after the targeted training approach.

	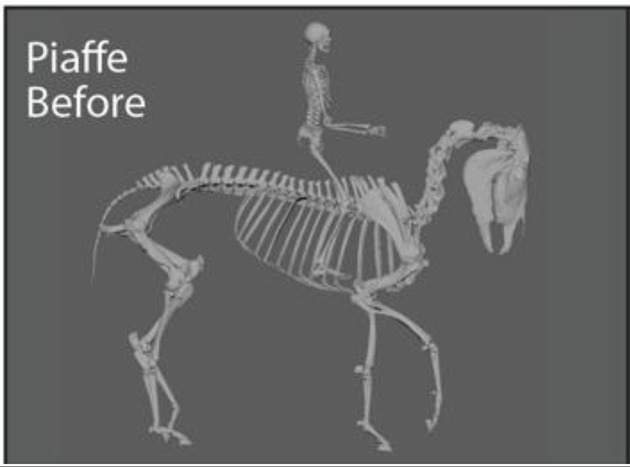		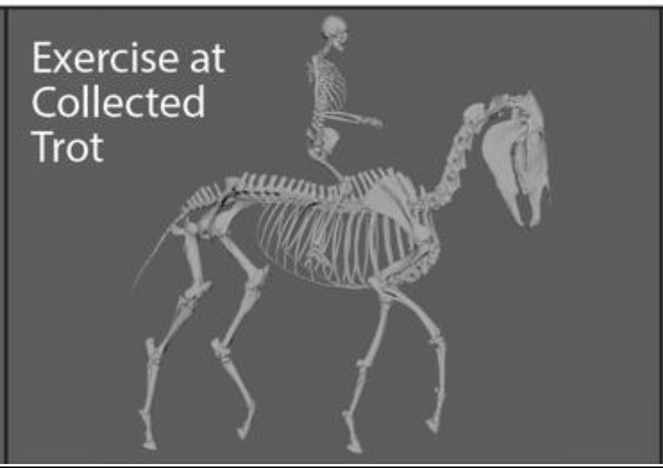		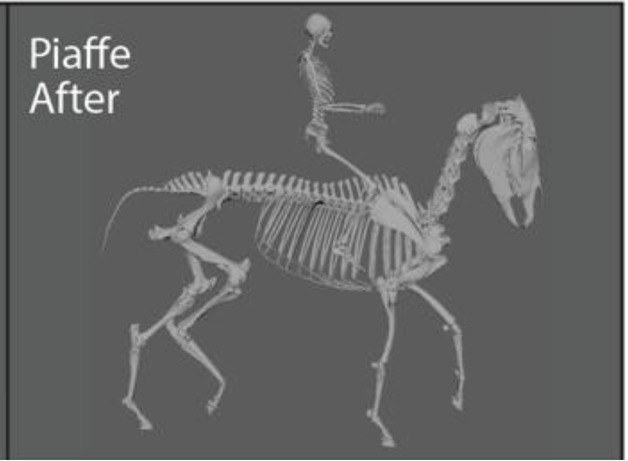	
	Weightbearing	Non-weightbearing	Weightbearing	Non-weightbearing	Weightbearing	Non-weightbearing
*Joint angles measured*						
**Pelvic limb**						
Hip angle	98.71°	78.80°	131.96°	89.96°	117.35°	82.27°
Stifle angle	119.30°	88.40°	132.87°	107.48°	113.24°	71.33°
Hock angle	149.20°	109.65°	146.70°	111.19°	133.25°	80.00°
Fetlock angle	238.40°	129.53°	209.80°	123.17°	229.13°	109.47°
**Thoracic limb**						
Shoulder angle	85.40°	95.60°	115.42°	82.75°	76.35°	73.20°
Elbow angle	115.40°	65.30°	145.18°	73.70°	122.14°	76.35°
Carpal angle	176.80°	127.02°	177.60°	127.10°	171.60°	135.35°
Fetlock angle	229.50°	161.50°	224.45°	172.05°	225.90°	176.70°
**Head and neck**						
Head–neck angle	113.90°		144.60°		149.90°	
Deviation of head from vertical	0.90° above the vertical		10.05° above the vertical		14.49° above the vertical	
*Intervertebral distances measured*						
C7 ➔ T1	3.22 cm		7.62 cm		4.78 cm	
T1 ➔ T2	11.63 cm		12.29 cm		11.05 cm	
T2 ➔ T3	5.94 cm		4.28 cm		4.14 cm	
T3 ➔ T4	5.95 cm		5.99 cm		5.97 cm	

## 3 Results

The results illustrate functionally relevant differences in joint angle values between the three positions (Tables 1, 2). Specifically, they identify reduction in the extreme fetlock joint angles that characterized the posture of the horse before therapy. These preliminary findings suggest that consistent exercise at the collected trot resulted in notable changes in the routine working posture of the horse at Piaffe (Tables 1, 2).

**Table 2:** Differences* in joint angle values and intervertebral distances before and after the targeted training approach.

	Weightbearing	Non-Weightbearing
*Joint angle differences*		
**Pelvic limb**		
Hip angle	−18.64°	−3.47°
Stifle angle	6.06°	17.07°
Hock angle	15.95°	29.65°
Fetlock angle	9.27°	20.06°
**Thoracic limb**		
Shoulder angle	9.05°	22.40°
Elbow angle	−6.74°	−11.05°
Carpal angle	5.20°	−8.33°
Fetlock angle	3.60°	−15.20°
**Head and neck**		
Head–neck angle	−36.00°	
Deviation of head from vertical	−13.59°	
*Intervertebral distance differences*		
C7 ➔ T1	−1.56 cm	
T1 ➔ T2	0.58 cm	
T2 ➔ T3	1.80 cm	
T3 ➔ T4	−0.02 cm	

*Difference in values = Piaffe before – Piaffe after.

Positive values = Piaffe before values > Piaffe after values.

Negative values = Piaffe before values < Piaffe after values.

## 4 Discussion

### 4.1 Analysis and functional interpretation

This case used the 3D dynamic horse model to document the changes in whole-body posture in a horse with bilateral fetlock joint degeneration after completion of an exercise program. The functional interpretation is based on the response to the exercises that suggests the degeneration in the fetlock joints was functional hyperextension, as it increases the loads acting on the structures of the joint and is associated with degenerative lesions [see (24, 25)]. The therapeutic effects of the whole-body exercise regime were able to be observed qualitatively and quantitatively and included an increased head–neck angle and a skull raised well above the vertical plane, and decompression of the cervical and early thoracic intervertebral spaces. Decreased hyperextension of the fetlock as indicated by their angles in all weightbearing limbs was also documented. These preliminary findings support the working hypothesis that the hyperextension of the fetlocks resulted from functional compression of the cervical and thoracic spine and set the stage for follow-up studies.

### 4.2 Dynamic 3D equine model

The dynamic 3D equine model (23) was designed with the goal of creating a useful tool for researchers, clinicians, trainers, and riders. Each joint can be specifically manipulated and analyzed individually to address research questions in depth. Additionally, while current 2D systems require that the perspective of the animal be perfectly matched for comparisons of movement, our model has the benefit of allowing accurate modeling in natural environments because it is 3D and able to be rotated and analyzed from any perspective. The model has also been rigged so that it can easily model postures and can be animated to demonstrate whole-body movements for use by clinicians, trainers, and riders. This flexibility enables the model to be widely used to analyze horse movement, experiment with different postures and movements, develop movement therapies, and visually depict findings for a variety of audiences.

This case study serves as proof of concept for the efficacy of using such 3D models to depict horse posture and movement and objectively document the effects of training and therapy programs. Currently the active part of a lameness exam focuses on a relatively restricted analysis of a horse’s movement: at the walk and trot, occasionally at canter, lunged, or ridden, and on hard or soft substrate (26). While these parameters are important for diagnosing lameness, treatment relies on knowing what is causing the lameness, which is better identified by an in depth postural and functional analysis of how a horse is performing. As illustrated with this example, the dynamic 3D equine model can be used for such a whole-body analytical approach. It can thus facilitate the diagnosis and treatment of the root causes of lamenesses, as well as provide the kind of individualized information needed to improve performances and prevent lameness (i.e.: prehabilitation).

With regards to study limitations for this retrospective case study, the modeled postures were based solely on matching the graphic model to representative images. While the fetlock joint is very prominent and is clearly depicted within the representative images without surface markers, other joints and skeletal elements, like the vertebrae, are difficult to see with the naked eye and would require markers for accuracy. For this case, the distal joints of the limb were used as for primary matching between the horse in the images and the model, with the placement of the head and topline of the animal as secondary matches. The rest of the positioning relied on the rigging system of the model (23) that placed the other elements in their relative positions. Also, perspective distortion is unavoidable at certain camera angles when using a single 2D image. A set-up that uses multiple cameras to capture different planes resolves this issue but is difficult even with small animals (27), where its use is relatively common; it is extremely impractical when trying to capture a horse’s natural movement in its natural environment. However, the alignment of a freeze frame image from overhead footage captured by drone can be combined with an image captured from the ground for increased accuracy in modeling. Finally, the angles were given by the software and the calculations for their measurements (described above) were done without statistical analysis. These study limitations do not negate the utility of the model itself.

By nature of individual variability, not every subject horse will align anatomically perfectly with the model. This is an acceptable limitation, at least until individual full-body CT scans are routinely available. When they are, the rigging of our 3D full-body dynamic equine model (23) can be easily applied to a unique data set. In the meantime, our model can be scaled as a whole, and individual regions (e.g., leg or neck) or elements (e.g., skull or femur) can be resized to better match the horse being studied.

The functionality of our 3D full-body dynamic equine model has been established in preliminary studies, which have confirmed the value of whole-body analysis for identifying the sources of tissue overloading in cases of navicular disease, club foot, chronic, caudal cervical osteoarthritis, and the current case of fetlock degeneration. Importantly, we have also documented that successful rehabilitation in these cases was characterized by correction of the postural changes identified with our model as the causes of the overloading. These studies also revealed that the analyses are time- and labor-intensive and require specialized training. Our next step is to streamline this technology by linking movement data (i.e., points from surface markers) from a living horse to the corresponding points on our already developed 3D horse model so that the skeleton can be easily animated to model the movement of a horse in a video (28, 29); this will make the dynamic 3D equine horse more efficient and practical for research purposes and more widely useful to clinicians, trainers, and riders.

## 5 Conclusion

This case study demonstrates the feasibility and efficacy of using an accurate 3D model generated from CT data to analyze the relationship between whole-body working postures and the sites of degenerative tissue changes. The dynamic 3D horse model can be animated to replicate movements (i.e., from motion analysis videos) and positioned to depict postures. The dynamic 3D horse, as depicted in this case study, can be used for research purposes and as an educational tool to help veterinary students, riders, and trainers better understand the structure and function of the equine body. While this particular model is a horse, the graphic rigging system can be easily applied to other animals like the dog, for which there is also broad interest in designing re- and pre-habilitative therapies.

## Data Availability

The original contributions presented in the study are included in the article/Supplementary material, further inquiries can be directed to the corresponding author.

## Supplementary material

SUPPLEMENTARY FIGURE 1 Angles and landmarks of the pelvic limb joints. The limb positions were first matched to the representative images and then rotated into a lateral perspective for analysis.

SUPPLEMENTARY FIGURE 2 Angles and landmarks for the thoracic limb joints. The limb positions were first matched to the representative images and then rotated into a lateral perspective for analysis.

SUPPLEMENTARY FIGURE 3 Angles and landmarks for the head and cervical spine **(A)**. A distance measure tool was used to find the distance between several cervical and thoracic vertebrae **(B)**. The head and neck positions were first matched to the representative images and then rotated into a lateral perspective for analysis.
