# Supplementary figures and images for: The dynamic 3D horse: analyzing the relationship between whole body pathomechanics and joint degeneration in the fetlocks
Source: Front Vet Sci. 2026 Mar 11;13:1773617. doi: 10.3389/fvets.2026.1773617 (PMC13014753; doi:10.3389/fvets.2026.1773617)

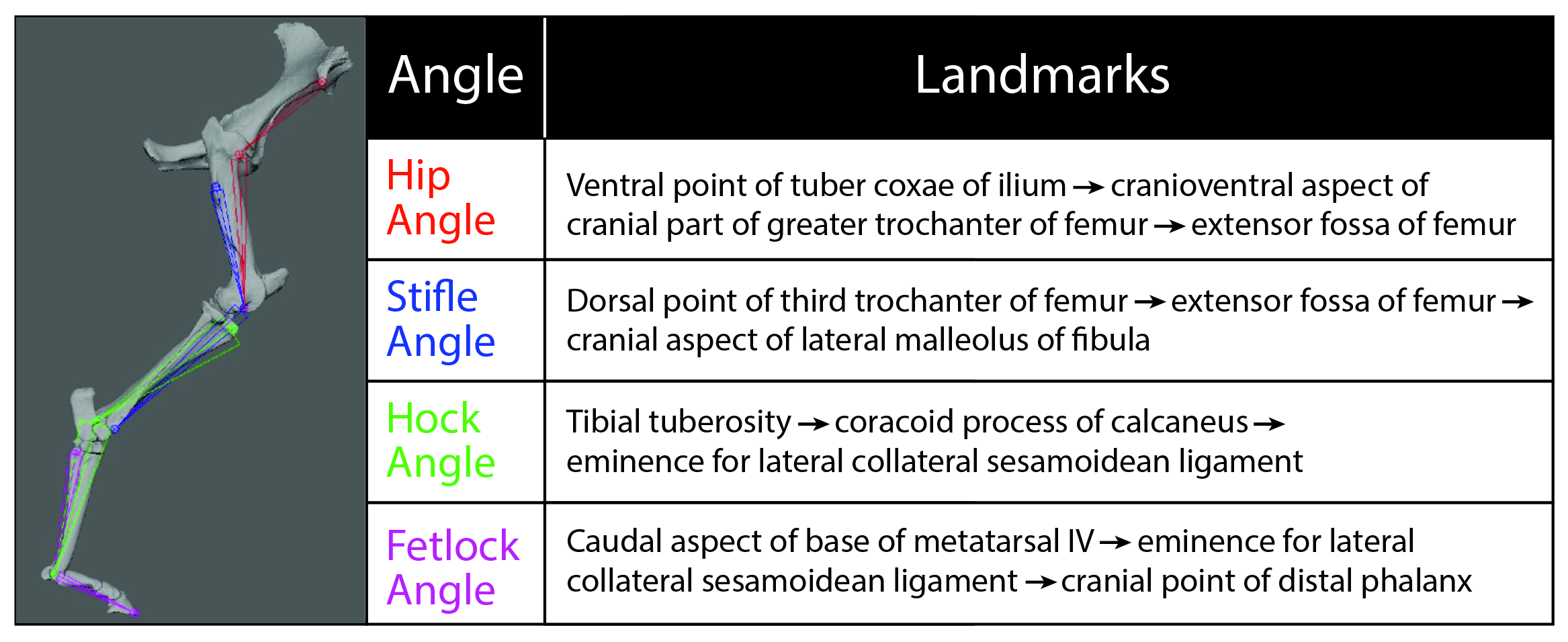

Supplement: SUPPLEMENTARY FIGURE 1 — Angles and landmarks of the pelvic limb joints. The limb positions were first matched to the representative images and then rotated into a lateral perspective for analysis. [file Image_1.tif]

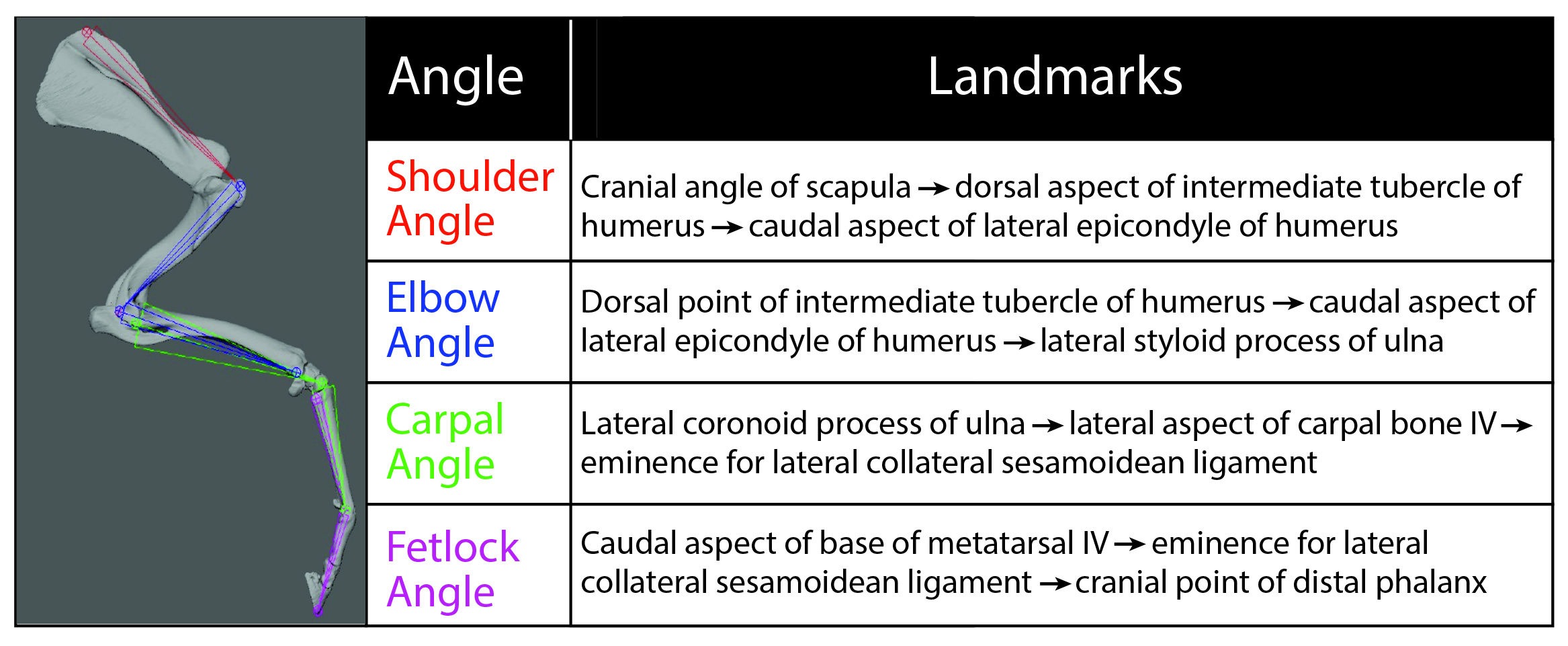

Supplement: SUPPLEMENTARY FIGURE 2 — Angles and landmarks for the thoracic limb joints. The limb positions were first matched to the representative images and then rotated into a lateral perspective for analysis. [file Image_2.tif]

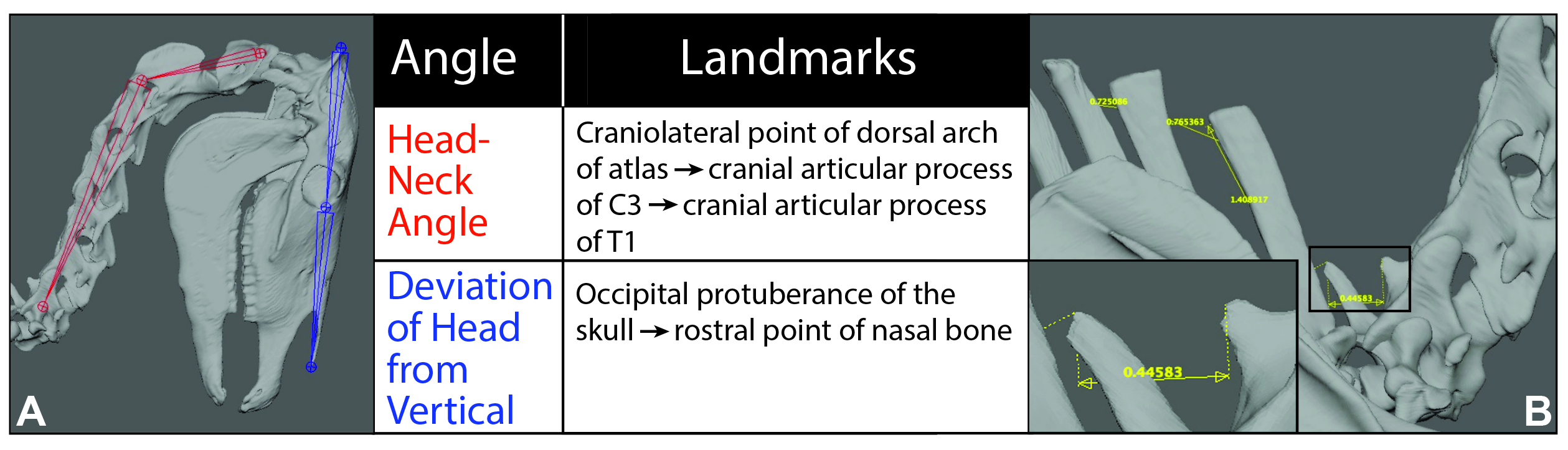

Supplement: SUPPLEMENTARY FIGURE 3 — Angles and landmarks for the head and cervical spine (A). A distance measure tool was used to find the distance between several cervical and thoracic vertebrae (B). The head and neck positions were first matched to the representative images and then rotated into a lateral perspective for analysis. [file Image_3.tif]
